# Supplementary material for: Hybrid Models and Biological Model Reduction with PyDSTool
Source: PLoS Comput Biol. 2012 Aug 9;8(8):e1002628. doi: 10.1371/journal.pcbi.1002628 (PMC3415397; doi:10.1371/journal.pcbi.1002628)
Supplement: Text S4 — Complete source code for the PyDSTool package (version 0.88.120504). Includes API documentation and help files linking to web pages. This file is identical to the current public release on Sourceforge.net. (ZIP) [file pcbi.1002628.s004.zip › PyDSTool/html/toc-PyDSTool.MProject-module.html]

xml version="1.0" encoding="ascii"?


MProject


# Module MProject

---

## Classes

GenTransform  
 GeneratorInterface  

MReg

ModelInterface  
 ModelLibrary  
 ModelManager  
 ModelTransform  
 always\_feature  
 binary\_feature  
 condition  
 context  

dsInterface

extModelInterface  
 feature  

feature\_leaf

feature\_node

intModelInterface  
 ql\_feature\_leaf  
 ql\_feature\_node  
 qt\_feature\_leaf  
 qt\_feature\_node  

## Functions

extract\_from\_model  

process\_raw\_residual

## Variables

\_classes

\_functions

---

[hide private]
